# Supplementary material for: Dynamics and Predictors of Mortality Due to Candidemia Caused by Different Candida Species: Comparison of Intensive Care Unit-Associated Candidemia (ICUAC) and Non-ICUAC
Source: J Fungi (Basel). 2021 Jul 24;7(8):597. doi: 10.3390/jof7080597 (PMC8397010; doi:10.3390/jof7080597)
Supplement: Supplementary file 1 [file jof-07-00597-s001.zip › Supplementary Table S1(A¿CE).pdf]

Supplementary Table S1A. Univariate analysis of predictive factors related to 7-, 30- and 90-day mortalities of all candidemia patients

	7-day mortality		30-day mortality		90-day mortality	
Variables	OR (95% CI)	*p*-Value	OR (95% CI)	*p*-Value	OR (95% CI)	*p*-Value
Aged 65 or older	1.49 (1.08-2.04)	0.01	1.30 (1.02-1.64)	0.03	1.30 (1.05-1.61)	0.02
Male	0.97 (0.72-1.31)	0.84	0.93 (0.74-1.17)	0.53	0.99 (0.80-1.23)	0.96
Community onset	0.20 (0.06-0.63)	0.006	0.40 (0.22-0.73)	0.003	0.56 (0.36-0.85)	0.006
ICU admission	2.58 (1.88-3.53)	<0.001	2.31 (1.82-2.92)	<0.001	2.27 (1.83-2.82)	<0.001
Diabetes mellitus	1.19 (0.87-1.63)	0.28	1.10 (0.86-1.41)	0.43	1.09 (0.87-1.37)	0.45
Liver disease	1.78 (1.23-2.56)	0.002	1.78 (1.34-2.38)	<0.001	1.77 (1.36-2.32)	<0.001
Chronic kidney disease	1.24 (0.83-1.84)	0.29	1.35 (1.00-1.81)	0.05	1.42 (1.08-1.85)	0.01
Chronic obstructive pulmonary disease	1.69 (1.10-2.60)	0.02	1.62 (1.16-2.27)	0.005	1.70 (1.26-2.31)	0.001
Congestive heart failure	1.03 (0.54-1.95)	0.93	1.40 (0.90-2.16)	0.13	1.48 (1.00-2.19)	0.05
Myocardial infarction	1.65 (0.97-2.79)	0.07	1.36 (0.88-2.11)	0.16	1.22 (0.81-1.85)	0.34
Cerebro-vascular disease or transient ischemic attack	0.91 (0.57-1.45)	0.69	0.93 (0.65-1.32)	0.68	1.03 (0.76-1.41)	0.85
Dementia	1.42 (0.80-2.49)	0.23	1.39 (0.89-2.17)	0.15	1.34 (0.89-2.03)	0.17
Solid tumor	1.03 (0.76-1.40)	0.84	0.94 (0.74-1.19)	0.61	0.97 (0.78-1.20)	0.77
Hematologic malignancies	1.44 (0.86-2.41)	0.17	1.43 (0.96-2.14)	0.08	1.41 (0.97-2.05)	0.07
ACCI more than 5	1.51 (1.10-2.06)	0.01	1.36 (1.07-1.72)	0.01	1.38 (1.11-1.71)	0.004
Severe sepsis	2.66 (1.98-3.59)	<0.001	2.76 (2.19-3.48)	<0.001	2.99 (2.42-3.69)	<0.001
Bacteremia	1.20 (0.84-1.71)	0.31	1.35 (1.03-1.76)	0.03	1.49 (1.18-1.90)	0.001
Total parenteral nutrition	1.14 (0.84-1.54)	0.39	1.26 (1.00-1.58)	0.05	1.34 (1.09-1.66)	0.006
Prior surgery	0.66 (0.43-1.03)	0.07	0.89 (0.66-1.20)	0.44	0.91 (0.69-1.20)	0.50
Neutropenia	1.10 (0.68-1.77)	0.70	1.28 (0.90-1.82)	0.16	1.28 (0.92-1.77)	0.14
Immunosuppressive therapy	0.73 (0.36-1.48)	0.39	1.14 (0.73-1.78)	0.56	0.99 (0.64-1.53)	0.97
Urine catheter placement	2.70 (1.91-3.80)	<0.001	2.77 (2.14-3.59)	<0.001	2.96 (2.33-3.76)	<0.001
CVC placement	2.14 (1.52-3.01)	<0.001	1.89 (1.47-2.43)	<0.001	1.92 (1.53-2.42)	<0.001
Prior fungal therapy	1.00 (0.54-1.85)	0.99	1.02 (0.64-1.62)	0.93	1.02 (0.67-1.56)	0.92
CVC removal	0.18 (0.11-0.30)	<0.001	0.46 (0.32-0.64)	<0.001	0.44 (0.32-0.61)	<0.001
Azole monotherapy	0.16 (0.09-0.27)	<0.001	0.41 (0.31-0.55)	<0.001	0.45 (0.35-0.58)	<0.001
Non-azole therapy	0.17 (0.11-0.29)	<0.001	0.52 (0.40-0.68)	<0.001	0.66 (0.53-0.83)	<0.001
Lack of antifungal therapy	13.10 (8.82-19.45)	<0.001	4.03 (3.20-5.09)	<0.001	3.18 (2.57-3.93)	<0.001
Fluconazole resistance	1.29 (0.70-2.37)	0.42	1.55 (0.99-2.41)	0.05	1.49 (0.98-2.25)	0.06
Micafungin resistance	0.05 (0.00-1,431.41)	0.57	0.78 (0.11-5.57)	0.81	0.77 (0.11-5.50)	0.80
Candidemia due to *C. albicans*	1.05 (0.77-1.41)	0.77	1.02 (0.81-1.28)	0.90	1.07 (0.86-1.32)	0.55
Candidemia due to *C. tropicalis*	1.67 (1.19-2.34)	0.003	1.51 (1.15-1.98)	0.003	1.59 (1.24-2.03)	<0.001
Candidemia due to *C. glabrata*	0.85 (0.57-1.28)	0.44	0.91 (0.67-1.23)	0.55	0.93 (0.71-1.23)	0.61

Candidemia due to *C. parapsilosis*	0.53 (0.31-0.91)	0.02	0.52 (0.35-0.78)	0.002	0.46 (0.31-0.67)	<0.001
Uncommon species	0.65 (0.29-1.47)	0.30	1.07 (0.65-1.77)	0.79	0.96 (0.59-1.56)	0.87
Multiple species	1.17 (0.48-2.85)	0.73	1.26 (0.65-2.45)	0.50	1.06 (0.55-2.06)	0.86

Abbreviations: OR, odds ratio; 95% CI, 95% confidence interval; ICU, intensive care unit; ACCI, age-adjusted Charlson comorbidity index; CVC, central venous catheter.

Supplementary Table S1B. Univariate analysis of predictive factors related to 7-, 30- and 90-day mortalities of candidemia patients due to *Candida albicans*

	7-day mortality		30-day mortality		90-day mortality	
Variables	OR (95% CI)	*p*-Value	OR (95% CI)	*p*-Value	OR (95% CI)	*p*-Value
Aged 65 or older	1.53 (0.93-2.50)	0.09	1.31 (0.91-1.89)	0.15	1.41 (1.01-1.98)	0.05
Male	1.13 (0.71-1.79)	0.62	1.06 (0.74-1.51)	0.76	1.14 (0.82-1.58)	0.43
Community onset	0.19 (0.03-1.36)	0.10	0.56 (0.23-1.38)	0.21	0.56 (0.25-1.26)	0.16
ICU admission	2.28 (1.42-3.64)	0.001	1.96 (1.37-2.80)	<0.001	2.00 (1.45-2.77)	<0.001
Diabetes mellitus	1.26 (0.78-2.03)	0.35	1.13 (0.77-1.64)	0.54	1.15 (0.82-1.62)	0.42
Liver disease	1.73 (0.99-3.01)	0.05	1.70 (1.10-2.65)	0.02	1.68 (1.12-2.52)	0.01
Chronic kidney disease	1.12 (0.59-2.13)	0.72	1.25 (0.77-2.01)	0.37	1.33 (0.87-2.04)	0.18
Chronic obstructive pulmonary disease	2.11 (1.14-3.91)	0.02	2.03 (1.24-3.31)	0.005	2.01 (1.29-3.14)	0.002
Congestive heart failure	0.92 (0.34-2.53)	0.88	0.98 (0.46-2.11)	0.96	1.18 (0.62-2.24)	0.62
Myocardial infarction	0.05 (0.00-8.04)	0.24	0.54 (0.17-1.70)	0.29	0.74 (0.30-1.80)	0.51
Cerebro-vascular disease or transient ischemic attack	1.06 (0.53-2.12)	0.88	0.96 (0.55-1.68)	0.90	1.08 (0.67-1.75)	0.76
Dementia	1.63 (0.75-3.54)	0.22	1.27 (0.64-2.50)	0.49	1.29 (0.70-2.39)	0.41
Solid tumor	1.12 (0.71-1.76)	0.63	1.01 (0.71-1.44)	0.95	1.06 (0.77-1.46)	0.74
Hematologic malignancies	1.78 (0.82-3.88)	0.15	1.22 (0.60-2.51)	0.58	0.97 (0.48-1.99)	0.94
ACCI more than 5	1.37 (0.84-2.22)	0.21	1.19 (0.82-1.71)	0.36	1.23 (0.88-1.72)	0.22
Severe sepsis	2.43 (1.54-3.84)	<0.001	2.77 (1.94-3.95)	<0.001	2.98 (2.15-4.12)	<0.001
Bacteremia	1.21 (0.70-2.08)	0.49	1.39 (0.92-2.09)	0.11	1.44 (0.99-2.08)	0.05
Total parenteral nutrition	1.12 (0.71-1.78)	0.62	1.29 (0.91-1.84)	0.16	1.44 (1.04-1.98)	0.03
Prior surgery	0.41 (0.19-0.90)	0.03	0.72 (0.44-1.16)	0.17	0.75 (0.49-1.15)	0.19
Neutropenia	1.13 (0.56-2.26)	0.74	1.23 (0.73-2.08)	0.44	1.17 (0.72-1.92)	0.53
Immunosuppressive therapy	0.49 (0.15-1.56)	0.23	0.87 (0.44-1.71)	0.68	0.69 (0.35-1.35)	0.28
Urine catheter use	2.59 (1.55-4.33)	<0.001	2.71 (1.82-4.02)	<0.001	2.95 (2.06-4.24)	<0.001
CVC use	2.55 (1.47-4.44)	0.001	2.25 (1.50-3.39)	<0.001	2.37 (1.63-3.44)	<0.001
Prior fungal therapy	0.87 (0.32-2.39)	0.79	1.01 (0.49-2.07)	0.98	0.90 (0.46-1.76)	0.75
CVC removal	0.39 (0.20-0.75)	0.005	0.44 (0.27-0.73)	0.001	0.39 (0.25-0.62)	<0.001
Azole monotherapy	0.12 (0.05-0.27)	<0.001	0.34 (0.22-0.53)	<0.001	0.41 (0.28-0.59)	<0.001
Non-azole therapy	0.16 (0.06-0.40)	<0.001	0.53 (0.34-0.83)	0.005	0.67 (0.46-0.98)	0.04
Lack of antifungal therapy	16.57 (8.70-31.55)	<0.001	4.84 (3.37-6.96)	<0.001	3.69 (2.66-5.11)	<0.001
Fluconazole resistance	0.05 (0.00-2,235,849.02)	0.74	3.53 (0.49-25.38)	0.21	3.53 (0.49-25.38)	0.21
Micafungin resistance	-	-	-	-	-	-

Abbreviations: OR, odds ratio; 95% CI, 95% confidence interval; ICU, intensive care unit; ACCI, age-adjusted Charlson comorbidity index; CVC, central venous catheter.

Supplementary Table S1C. Univariate analysis of predictive factors related to 7-, 30- and 90-day mortalities of candidemia patients due to *Candida tropicalis*

	7-day mortality		30-day mortality		90-day mortality	
Variables	OR (95% CI)	*p*-Value	OR (95% CI)	*p*-Value	OR (95% CI)	*p*-Value
Aged 65 or older	1.17 (0.66-2.09)	0.59	1.21 (0.75-1.93)	0.44	1.09 (0.71-1.66)	0.71
Male	1.08 (0.60-1.95)	0.81	1.05 (0.65-1.69)	0.86	1.08 (0.70-1.68)	0.72
Community onset	1.02 (0.25-4.22)	0.98	0.63 (0.15-2.57)	0.52	0.53 (0.13-2.16)	0.38
ICU admission	1.77 (0.95-3.28)	0.07	1.97 (1.19-3.26)	0.009	2.05 (1.29-3.23)	0.002
Diabetes mellitus	0.81 (0.41-1.60)	0.55	1.07 (0.64-1.79)	0.79	0.86 (0.52-1.41)	0.54
Liver disease	1.38 (0.70-2.72)	0.35	1.59 (0.93-2.72)	0.09	1.69 (1.03-2.77)	0.04
Chronic kidney disease	1.00 (0.47-2.14)	1.00	1.56 (0.91-2.70)	0.11	1.60 (0.96-2.68)	0.07
Chronic obstructive pulmonary disease	1.18 (0.46-2.98)	0.73	1.06 (0.48-2.31)	0.89	1.21 (0.61-2.42)	0.59
Congestive heart failure	1.83 (0.72-4.62)	0.20	2.38 (1.18-4.81)	0.02	2.05 (1.02-4.09)	0.04
Myocardial infarction	1.50 (0.59-3.80)	0.39	1.36 (0.62-2.97)	0.44	1.12 (0.52-2.43)	0.77
Cerebro-vascular disease or transient ischemic attack	0.54 (0.17-1.74)	0.30	0.56 (0.23-1.40)	0.22	0.62 (0.29-1.35)	0.23
Dementia	0.81 (0.20-3.34)	0.77	1.39 (0.56-3.44)	0.48	1.11 (0.45-2.73)	0.83
Solid tumor	0.73 (0.38-1.40)	0.34	0.82 (0.49-1.37)	0.44	0.88 (0.55-1.40)	0.58
Hematologic malignancies	1.17 (0.52-2.63)	0.70	1.25 (0.66-2.39)	0.49	1.25 (0.69-2.25)	0.46
ACCI more than 5	1.23 (0.68-2.22)	0.50	1.44 (0.89-2.36)	0.14	1.53 (0.98-2.39)	0.06
Severe sepsis	2.76 (1.54-4.95)	0.001	2.81 (1.75-4.50)	<0.001	3.07 (1.99-4.74)	<0.001
Bacteremia	1.00 (0.51-1.97)	1.00	1.06 (0.61-1.83)	0.84	1.29 (0.80-2.08)	0.30
Total parenteral nutrition	1.09 (0.61-1.94)	0.78	1.05 (0.65-1.68)	0.85	1.11 (0.72-1.70)	0.64
Prior surgery	0.84 (0.39-1.80)	0.65	0.87 (0.48-1.60)	0.66	0.84 (0.49-1.46)	0.54
Neutropenia	1.37 (0.61-3.07)	0.44	1.79 (0.96-3.35)	0.07	1.78 (0.98-3.24)	0.06
Immunosuppressive therapy	0.59 (0.14-2.45)	0.47	1.00 (0.40-2.47)	0.99	0.78 (0.32-1.94)	0.60
Urine catheter placement	2.86 (1.45-5.63)	0.002	2.87 (1.67-4.91)	<0.001	3.29 (2.01-5.41)	<0.001
CVC placement	1.82 (0.98-3.36)	0.06	1.70 (1.04-2.77)	0.04	1.78 (1.14-2.78)	0.01
Prior fungal therapy	0.36 (0.05-2.64)	0.32	0.50 (0.12-2.04)	0.33	0.61 (0.19-1.92)	0.39
CVC removal	0.40 (0.15-1.05)	0.06	0.64 (0.32-1.26)	0.20	0.61 (0.32-1.13)	0.12
Azole monotherapy	0.32 (0.13-0.82)	0.02	0.51 (0.27-0.95)	0.03	0.46 (0.26-0.81)	0.007
Non-azole therapy	0.32 (0.16-0.64)	0.001	0.52 (0.32-0.85)	0.009	0.67 (0.44-1.04)	0.07
Lack of antifungal therapy	6.43 (3.48-11.89)	<0.001	3.69 (2.29-5.93)	<0.001	3.14 (2.03-4.85)	<0.001
Fluconazole resistance	1.72 (0.24-12.47)	0.59	1.14 (0.16-8.21)	0.90	1.92 (0.47-7.83)	0.36
Micafungin resistance	0.05 (0.00-91,794.28)	0.68	0.05 (0.00-2,457.45)	0.59	0.05 (0.00-2,457.45)	0.59

Abbreviations: OR, odds ratio; 95% CI, 95% confidence interval; ICU, intensive care unit; ACCI, age-adjusted Charlson comorbidity index; CVC, central venous catheter.

Supplementary Table S1D. Univariate analysis of predictive factors related to 7-, 30- and 90-day mortalities of candidemia patients due to *Candida glabrata*

	7-day mortality		30-day mortality		90-day mortality	
Variables	OR (95% CI)	*p*-Value	OR (95% CI)	*p*-Value	OR (95% CI)	*p*-Value
Aged 65 or older	1.67 (0.68-4.13)	0.26	1.03 (0.57-1.86)	0.92	1.07 (0.62-1.83)	0.82
Male	1.17 (0.56-2.48)	0.67	1.06 (0.61-1.84)	0.84	1.18 (0.71-1.96)	0.53
Community onset	0.04 (0.00-3.37)	0.15	0.41 (0.15-1.13)	0.09	0.51 (0.22-1.19)	0.12
ICU admission	3.74 (1.69-8.28)	0.001	2.73 (1.56-4.76)	<0.001	2.50 (1.51-4.14)	<0.001
Diabetes mellitus	1.41 (0.66-3.01)	0.37	0.79 (0.43-1.47)	0.46	1.01 (0.59-1.72)	0.98
Liver disease	1.99 (0.81-4.91)	0.14	1.84 (0.92-3.68)	0.08	1.66 (0.86-3.19)	0.13
Chronic kidney disease	3.15 (1.42-6.98)	0.005	1.64 (0.82-3.27)	0.16	1.78 (0.96-3.29)	0.07
Chronic obstructive pulmonary disease	1.87 (0.71-4.93)	0.20	1.68 (0.79-3.58)	0.18	1.77 (0.90-3.49)	0.10
Congestive heart failure	0.80 (0.11-5.91)	0.83	1.39 (0.43-4.46)	0.58	1.55 (0.56-4.28)	0.40
Myocardial infarction	2.76 (0.96-7.97)	0.06	1.83 (0.73-4.62)	0.20	1.47 (0.59-3.67)	0.41
Cerebro-vascular disease or transient ischemic attack	1.56 (0.63-3.85)	0.33	1.67 (0.86-3.25)	0.13	1.67 (0.91-3.09)	0.10
Dementia	1.90 (0.57-6.28)	0.30	1.89 (0.75-4.76)	0.18	1.90 (0.82-4.42)	0.14
Solid tumor	1.16 (0.55-2.44)	0.70	0.96 (0.55-1.67)	0.88	0.90 (0.54-1.50)	0.69
Hematologic malignancies	1.08 (0.26-4.56)	0.92	2.02 (0.86-4.75)	0.11	2.11 (0.96-4.63)	0.06
ACCI more than 5	1.85 (0.78-4.35)	0.16	1.16 (0.65-2.08)	0.62	0.99 (0.59-1.67)	0.97
Severe sepsis	1.96 (0.92-4.18)	0.08	2.03 (1.15-3.55)	0.01	2.43 (1.46-4.04)	0.001
Bacteremia	1.98 (0.92-4.30)	0.08	2.31 (1.31-4.08)	0.004	2.39 (1.43-4.02)	0.001
Total parenteral nutrition	1.83 (0.87-3.85)	0.11	1.60 (0.92-2.78)	0.10	1.67 (1.01-2.77)	0.05
Prior surgery	0.83 (0.29-2.39)	0.73	1.22 (0.61-2.45)	0.57	1.26 (0.67-2.36)	0.48
Neutropenia	0.41 (0.06-2.99)	0.38	0.95 (0.34-2.63)	0.92	1.05 (0.42-2.64)	0.91
Immunosuppressive therapy	1.01 (0.14-7.45)	0.99	1.05 (0.26-4.33)	0.94	0.83 (0.20-3.41)	0.80
Urine catheter placement	3.09 (1.31-7.27)	0.01	2.54 (1.40-4.59)	0.002	2.54 (1.48-4.34)	0.001
CVC placement	2.86 (1.16-7.06)	0.02	1.97 (1.08-3.61)	0.03	1.66 (0.97-2.83)	0.07
Prior fungal therapy	1.17 (0.28-4.92)	0.83	0.96 (0.30-3.07)	0.94	1.06 (0.39-2.93)	0.91
CVC removal	0.20 (0.03-1.49)	0.12	0.61 (0.23-1.57)	0.30	0.91 (0.42-2.00)	0.82
Azole monotherapy	0.04 (0.00-4.92)	0.19	0.73 (0.29-1.85)	0.51	0.62 (0.25-1.54)	0.30
Non-azole therapy	0.03 (0.00-0.24)	0.001	0.32 (0.17-0.58)	<0.001	0.49 (0.29-0.82)	0.006
Lack of antifungal therapy	53.11 (7.21-391.38)	<0.001	3.55 (2.02-6.26)	<0.001	2.54 (1.53-4.21)	<0.001
Fluconazole resistance	2.81 (0.97-8.12)	0.06	2.63 (1.12-6.19)	0.03	2.58 (1.17-5.68)	0.02
Micafungin resistance	0.05 (0.00-34,952.09)	0.66	1.39 (0.19-10.04)	0.75	1.39 (0.19-10.04)	0.75

Abbreviations: OR, odds ratio; 95% CI, 95% confidence interval; ICU, intensive care unit; ACCI, age-adjusted Charlson comorbidity index; CVC, central venous catheter.

Supplementary Table S1E. Univariate analysis of predictive factors related to 7-, 30- and 90-day mortalities of candidemia patients due to *Candida parapsilosis*

	7-day mortality		30-day mortality		90-day mortality	
Variables	OR (95% CI)	*p*-Value	OR (95% CI)	*p*-Value	OR (95% CI)	*p*-Value
Aged 65 or older	3.85 (1.07-13.80)	0.04	2.32 (1.00-5.37)	0.05	2.36 (1.07-5.22)	0.03
Male	0.37 (0.13-1.05)	0.06	0.54 (0.24-1.19)	0.13	0.65 (0.30-1.40)	0.27
Community onset	0.04 (0.00-156.94)	0.45	0.04 (0.00-15.00)	0.29	0.04 (0.00-10.07)	0.26
ICU admission	3.53 (1.11-11.27)	0.03	3.71 (1.55-8.87)	0.003	3.61 (1.59-8.19)	0.002
Diabetes mellitus	0.74 (0.21-2.66)	0.65	1.07 (0.45-2.56)	0.88	1.11 (0.49-2.51)	0.81
Liver disease	1.20 (0.16-9.20)	0.86	1.84 (0.92-3.68)	0.08	0.60 (0.08-4.46)	0.62
Chronic kidney disease	0.56 (0.07-4.24)	0.57	0.59 (0.14-2.52)	0.48	0.81 (0.25-2.69)	0.74
Chronic obstructive pulmonary disease	0.85 (0.11-6.49)	0.87	2.17 (0.74-6.32)	0.16	2.59 (0.98-6.83)	0.05
Congestive heart failure	0.05 (0.00-942.76)	0.54	1.37 (0.32-5.82)	0.67	1.93 (0.58-6.39)	0.28
Myocardial infarction	4.04 (1.12-14.53)	0.03	2.08 (0.62-6.96)	0.24	1.78 (0.54-5.92)	0.34
Cerebro-vascular disease or transient ischemic attack	0.39 (0.05-2.95)	0.36	0.66 (0.20-2.22)	0.51	1.07 (0.41-2.82)	0.89
Dementia	0.05 (0.00-1,173,828.13)	0.73	2.19 (0.30-16.21)	0.44	1.99 (0.27-14.65)	0.50
Solid tumor	1.05 (0.35-3.13)	0.93	0.76 (0.32-1.81)	0.53	0.78 (0.34-1.78)	0.56
Hematologic malignancies	0.05 (0.00-1,270,941,830.07)	0.81	5.50 (0.73-41.20)	0.10	5.50 (0.73-41.20)	0.10
ACCI more than 5	1.75 (0.62-5.00)	0.29	1.42 (0.64-3.13)	0.39	1.62 (0.77-3.41)	0.20
Severe sepsis	3.93 (1.36-11.34)	0.01	3.69 (1.65-8.21)	0.001	3.78 (1.76-8.09)	0.001
Bacteremia	0.04 (0.00-53.53)	0.38	0.62 (0.15-2.62)	0.52	0.85 (0.26-2.82)	0.79
Total parenteral nutrition	1.33 (0.47-3.80)	0.59	1.71 (0.78-3.76)	0.18	1.56 (0.74-3.28)	0.24
Prior surgery	0.85 (0.19-3.81)	0.84	0.93 (0.32-2.72)	0.90	1.07 (0.41-2.81)	0.89
Neutropenia	0.85 (0.11-6.49)	0.87	0.95 (0.22-4.03)	0.94	0.82 (0.19-3.46)	0.79
Immunosuppressive therapy	2.81 (0.37-21.48)	0.32	1.60 (0.22-11.84)	0.65	1.39 (0.19-10.26)	0.75
Urine catheter placement	1.74 (0.58-5.20)	0.32	3.13 (1.25-7.83)	0.02	3.70 (1.50-9.14)	0.004
CVC placement	1.47 (0.49-4.39)	0.49	1.48 (0.66-3.36)	0.34	1.76 (0.80-3.89)	0.16
Prior fungal therapy	3.33 (0.74-14.94)	0.12	1.97 (0.46-8.37)	0.36	1.73 (0.41-7.29)	0.46
CVC removal	0.16 (0.02-1.25)	0.08	0.37 (0.12-1.14)	0.08	0.27 (0.09-0.83)	0.02
Azole monotherapy	0.07 (0.01-0.54)	0.01	0.33 (0.14-0.78)	0.01	0.46 (0.21-0.99)	0.05
Non-azole therapy	0.03 (0.00-7.78)	0.22	0.46 (0.14-1.54)	0.21	0.40 (0.12-1.34)	0.14
Lack of antifungal therapy	37.22 (4.86-285.12)	<0.001	5.14 (2.30-11.51)	<0.001	4.12 (1.95-8.73)	<0.001
Fluconazole resistance	0.05 (0.00-8,416.98)	0.62	1.10 (0.15-8.15)	0.93	1.00 (0.14-7.35)	1.00
Micafungin resistance	-	-	-	-	-	-

Abbreviations: OR, odds ratio; 95% CI, 95% confidence interval; ICU, intensive care unit; ACCI, age-adjusted Charlson comorbidity index; CVC, central venous catheter.
