# Supplementary material for: Dynamics and Predictors of Mortality Due to Candidemia Caused by Different Candida Species: Comparison of Intensive Care Unit-Associated Candidemia (ICUAC) and Non-ICUAC
Source: J Fungi (Basel). 2021 Jul 24;7(8):597. doi: 10.3390/jof7080597 (PMC8397010; doi:10.3390/jof7080597)
Supplement: Supplementary file 1 [file jof-07-00597-s001.zip › Supplementary Table S2(A¿CD).pdf]

Supplementary Table S2A. Univariate analysis of predictive factors related to 7-, 30- and 90-day mortalities of ICUAC and non-ICUAC due to *Candida albicans*

		7-day mortality		30-day mortality		90-day mortality	
Variables	Setting	OR (95% CI)	*p*-Value	OR (95% CI)	*p*-Value	OR (95% CI)	*p*-Value
Aged 65 or older	ICUAC	1.65 (0.85-3.19)	0.14	1.29 (0.78-2.13)	0.32	1.54 (0.97-2.44)	0.07
	Non-ICUAC	1.20 (0.56-2.56)	0.64	1.20 (0.69-2.09)	0.51	1.18 (0.72-1.94)	0.52
Male	ICUAC	0.71 (0.40-1.27)	0.25	0.87 (0.54-1.39)	0.56	0.97 (0.63-1.50)	0.89
	Non-ICUAC	1.97 (0.89-4.36)	0.09	1.17 (0.68-2.01)	0.57	1.19 (0.73-1.94)	0.50
Community onset	ICUAC	-	-	-	-	-	-
	Non-ICUAC	0.28 (0.04-2.08)	0.22	0.79 (0.31-1.98)	0.61	0.79 (0.34-1.83)	0.58
Diabetes mellitus	ICUAC	1.30 (0.71-2.36)	0.39	1.08 (0.66-1.78)	0.76	1.20 (0.77-1.87)	0.42
	Non-ICUAC	1.13 (0.51-2.51)	0.76	1.14 (0.64-2.04)	0.65	1.06 (0.62-1.80)	0.83
Liver disease	ICUAC	1.39 (0.69-2.81)	0.35	1.59 (0.91-2.78)	0.10	1.64 (0.98-2.74)	0.06
	Non-ICUAC	2.04 (0.83-5.03)	0.12	1.61 (0.79-3.31)	0.19	1.48 (0.76-2.91)	0.25
Chronic kidney disease	ICUAC	1.53 (0.74-3.17)	0.25	1.38 (0.75-2.52)	0.30	1.48 (0.86-2.56)	0.16
	Non-ICUAC	0.52 (0.12-2.17)	0.37	1.00 (0.45-2.21)	1.00	1.16 (0.59-2.29)	0.66
Chronic obstructive pulmonary disease	ICUAC	2.51 (1.25-5.07)	0.01	2.09 (1.15-3.83)	0.02	2.29 (1.33-3.95)	0.003
	Non-ICUAC	1.00 (0.24-4.20)	1.00	1.60 (0.69-3.75)	0.28	1.48 (0.67-3.24)	0.33
Congestive heart failure	ICUAC	1.18 (0.42-3.29)	0.75	0.72 (0.26-1.97)	0.52	1.04 (0.48-2.26)	0.91
	Non-ICUAC	0.05 (0.00-121.59)	0.44	1.25 (0.39-3.99)	0.71	1.08 (0.34-3.43)	0.90
Myocardial infarction	ICUAC	0.05 (0.00-17.77)	0.31	0.25 (0.03-1.80)	0.17	0.37 (0.09-1.49)	0.16
	Non-ICUAC	0.05 (0.00-404.81)	0.51	1.07 (0.26-4.39)	0.93	1.42 (0.44-4.52)	0.56
Cerebro-vascular disease or transient ischemic attack	ICUAC	0.93 (0.44-2.00)	0.86	1.02 (0.56-1.86)	0.96	1.05 (0.61-1.81)	0.86
	Non-ICUAC	0.51 (0.07-3.78)	0.51	0.24 (0.03-1.74)	0.16	0.57 (0.18-1.81)	0.34
Dementia	ICUAC	1.40 (0.59-3.31)	0.44	1.24 (0.59-2.59)	0.57	1.38 (0.71-2.68)	0.34
	Non-ICUAC	1.07 (0.15-7.89)	0.95	0.52 (0.07-3.78)	0.52	0.41 (0.06-2.95)	0.38
Solid tumor	ICUAC	1.52 (0.84-2.77)	0.17	1.14 (0.68-1.90)	0.62	1.34 (0.85-2.11)	0.20
	Non-ICUAC	1.58 (0.70-3.59)	0.27	1.47 (0.83-2.62)	0.19	1.42 (0.84-2.39)	0.19

Hematologic malignancies	ICUAC	1.90 (0.81-4.49)	0.14	1.42 (0.65-3.09)	0.38	1.14 (0.52-2.47)	0.74
	Non-ICUAC	0.84 (0.11-6.19)	0.86	0.44 (0.06-3.19)	0.42	0.36 (0.05-2.58)	0.31
ACCI more than 5	ICUAC	1.63 (0.88-3.02)	0.12	1.23 (0.76-1.99)	0.39	1.40 (0.90-2.17)	0.14
	Non-ICUAC	1.20 (0.54-2.65)	0.65	1.27 (0.71-2.27)	0.41	1.20 (0.71-2.03)	0.49
Severe sepsis	ICUAC	1.89 (1.06-3.37)	0.03	2.26 (1.41-3.63)	0.001	2.56 (1.66-3.95)	<0.001
	Non-ICUAC	2.34 (1.06-5.17)	0.04	2.63 (1.47-4.68)	0.001	2.74 (1.62-4.62)	<0.001
Bacteremia	ICUAC	1.08 (0.57-2.05)	0.82	0.96 (0.56-1.64)	0.88	1.00 (0.62-1.61)	0.99
	Non-ICUAC	0.96 (0.33-2.78)	0.95	1.91 (1.02-3.57)	0.04	1.87 (1.05-3.34)	0.03
Total parenteral nutrition	ICUAC	1.00 (0.56-1.78)	0.99	0.93 (0.58-1.49)	0.76	1.13 (0.74-1.73)	0.57
	Non-ICUAC	1.23 (0.59-2.59)	0.58	1.83 (1.07-3.15)	0.03	1.73 (1.06-2.84)	0.03
Prior surgery	ICUAC	0.44 (0.18-1.03)	0.06	0.67 (0.37-1.21)	0.19	0.74 (0.44-1.23)	0.25
	Non-ICUAC	0.19 (0.03-1.41)	0.11	0.63 (0.27-1.47)	0.28	0.52 (0.22-1.20)	0.13
Neutropenia	ICUAC	1.55 (0.61-3.93)	0.35	1.27 (0.55-2.92)	0.58	1.27 (0.59-2.76)	0.54
	Non-ICUAC	1.06 (0.37-3.05)	0.92	1.49 (0.75-2.97)	0.25	1.37 (0.72-2.63)	0.34
Immunosuppressive therapy	ICUAC	0.74 (0.18-3.05)	0.68	1.31 (0.53-3.26)	0.56	1.09 (0.44-2.70)	0.85
	Non-ICUAC	0.36 (0.05-2.63)	0.31	0.72 (0.26-1.98)	0.52	0.56 (0.20-1.54)	0.26
Urine catheter placement	ICUAC	2.89 (1.23-6.83)	0.02	3.07 (1.52-6.18)	0.002	4.10 (2.11-7.96)	<0.001
	Non-ICUAC	1.66 (0.79-3.48)	0.18	1.95 (1.13-3.34)	0.02	1.94 (1.19-3.17)	0.008
CVC placement	ICUAC	2.90 (1.23-6.85)	0.02	3.00 (1.53-5.86)	0.001	3.12 (1.72-5.65)	<0.001
	Non-ICUAC	1.74 (0.81-3.78)	0.16	1.51 (0.87-2.62)	0.14	1.63 (0.98-2.70)	0.06
Prior fungal therapy	ICUAC	1.34 (0.48-3.73)	0.58	1.61 (0.74-3.53)	0.23	1.64 (0.79-3.40)	0.19
	Non-ICUAC	0.05 (0.00-53.37)	0.39	0.28 (0.04-2.03)	0.21	0.21 (0.03-1.50)	0.12
CVC removal	ICUAC	0.42 (0.19-0.91)	0.03	0.43 (0.23-0.80)	0.008	0.39 (0.22-0.69)	0.001
	Non-ICUAC	0.33 (0.10-1.16)	0.08	0.46 (0.20-1.08)	0.07	0.35 (0.16-0.80)	0.01
Azole monotherapy	ICUAC	0.11 (0.03-0.36)	<0.001	0.29 (0.15-0.54)	<0.001	0.37 (0.22-0.62)	<0.001
	Non-ICUAC	0.14 (0.04-0.48)	0.002	0.44 (0.24-0.80)	0.007	0.49 (0.29-0.83)	0.009
Non-azole therapy	ICUAC	0.13 (0.04-0.41)	0.001	0.42 (0.23-0.76)	0.004	0.55 (0.33-0.90)	0.02
	Non-ICUAC	0.20 (0.05-0.84)	0.03	0.64 (0.33-1.24)	0.19	0.77 (0.43-1.37)	0.37
Lack of antifungal therapy	ICUAC	20.20 (8.49-48.08)	<0.001	7.15 (4.32-11.84)	<0.001	5.47 (3.50-8.55)	<0.001

	Non-ICUAC	12.98 (4.92-34.24)	<0.001	3.37 (1.96-5.80)	<0.001	2.73 (1.66-4.48)	<0.001
Fluconazole resistance	ICUAC	-	-	-	-	-	-
	Non-ICUAC	0.05 (0.00-174,675,229.90)	0.79	5.12 (0.70-37.48)	0.11	5.12 (0.70-37.48)	0.11
Micafungin resistance	ICUAC	-	-	-	-	-	-
	Non-ICUAC	-	-	-	-	-	-

Abbreviations: ICUAC, intensive care unit associated candidemia; OR, odds ratio; 95% CI, 95% confidence interval; ACCI, age-adjusted Charlson comorbidity index; CVC, central venous catheter.

Supplementary Table S2B. Univariate analysis of predictive factors related to 7-, 30- and 90-day mortalities of ICUAC and non-ICUAC due to *Candida tropicalis*

		7-day mortality		30-day mortality		90-day mortality	
Variables	Setting	OR (95% CI)	*p*-Value	OR (95% CI)	*p*-Value	OR (95% CI)	*p*-Value
Aged 65 or older	ICUAC	1.31 (0.65-2.64)	0.46	1.81 (1.02-3.21)	0.04	1.66 (0.99-2.80)	0.06
	Non-ICUAC	1.12 (0.40-3.15)	0.83	0.74 (0.32-1.71)	0.49	0.68 (0.32-1.45)	0.32
Male	ICUAC	1.04 (0.51-2.12)	0.92	1.05 (0.59-1.87)	0.87	1.10 (0.65-1.86)	0.73
	Non-ICUAC	1.24 (0.42-3.64)	0.69	1.11 (0.47-2.65)	0.81	1.09 (0.50-2.37)	0.84
Community onset	ICUAC	-	-	-	-	-	-
	Non-ICUAC	1.53 (0.35-6.82)	0.57	0.98 (0.23-4.21)	0.98	0.82 (0.19-3.48)	0.79
Diabetes mellitus	ICUAC	1.01 (0.45-2.27)	0.97	1.61 (0.89-2.92)	0.11	1.39 (0.79-2.45)	0.26
	Non-ICUAC	0.55 (0.16-1.95)	0.36	0.47 (0.16-1.38)	0.17	0.36 (0.13-1.06)	0.06
Liver disease	ICUAC	1.73 (0.79-3.76)	0.17	1.86 (0.99-3.46)	0.05	2.13 (1.20-3.78)	0.01
	Non-ICUAC	0.70 (0.16-3.11)	0.64	1.05 (0.36-3.11)	0.93	0.90 (0.31-2.61)	0.85
Chronic kidney disease	ICUAC	0.91 (0.37-2.21)	0.83	1.57 (0.83-2.98)	0.17	1.65 (0.91-3.00)	0.10
	Non-ICUAC	0.99 (0.22-4.38)	0.99	1.35 (0.46-3.99)	0.59	1.18 (0.41-3.44)	0.76
Chronic obstructive pulmonary disease	ICUAC	0.88 (0.27-2.88)	0.83	0.89 (0.35-2.26)	0.81	1.11 (0.50-2.44)	0.80
	Non-ICUAC	1.80 (0.41-8.01)	0.44	1.21 (0.28-5.18)	0.80	1.08 (0.26-4.59)	0.91
Congestive heart failure	ICUAC	2.54 (0.88-7.27)	0.08	3.34 (1.49-7.52)	0.003	3.38 (1.50-7.59)	0.003
	Non-ICUAC	0.98 (0.13-7.42)	0.98	1.42 (0.33-6.08)	0.64	1.12 (0.26-4.72)	0.88
Myocardial infarction	ICUAC	1.75 (0.61-5.00)	0.30	1.65 (0.70-3.89)	0.25	1.40 (0.60-3.26)	0.44
	Non-ICUAC	0.98 (0.13-7.42)	0.98	0.64 (0.09-4.76)	0.66	0.51 (0.07-3.79)	0.51
Cerebro-vascular disease or transient ischemic attack	ICUAC	0.36 (0.05-2.61)	0.31	0.41 (0.10-1.70)	0.22	0.52 (0.16-1.66)	0.27
	Non-ICUAC	0.87 (0.20-3.87)	0.86	0.88 (0.26-2.99)	0.84	0.91 (0.31-2.64)	0.87
Dementia	ICUAC	0.48 (0.07-3.54)	0.47	1.41 (0.50-3.92)	0.52	1.23 (0.44-3.40)	0.69
	Non-ICUAC	1.68 (0.22-12.81)	0.62	1.06 (0.14-7.90)	0.95	0.76 (0.10-5.62)	0.79
Solid tumor	ICUAC	0.59 (0.24-1.45)	0.25	0.84 (0.44-1.62)	0.60	0.90 (0.50-1.62)	0.73
	Non-ICUAC	1.12 (0.40-3.14)	0.83	0.95 (0.40-2.26)	0.91	1.01 (0.46-2.21)	0.97

Hematologic malignancies	ICUAC	1.36 (0.52-3.56)	0.53	1.27 (0.57-2.84)	0.56	1.27 (0.60-2.69)	0.53
	Non-ICUAC	0.90 (0.20-4.00)	0.89	1.32 (0.45-3.89)	0.62	1.37 (0.52-3.62)	0.53
ACCI more than 5	ICUAC	1.29 (0.63-2.66)	0.49	1.82 (1.00-3.33)	0.05	2.21 (1.26-3.87)	0.006
	Non-ICUAC	1.10 (0.39-3.09)	0.86	1.03 (0.44-2.41)	0.95	0.92 (0.43-1.96)	0.83
Severe sepsis	ICUAC	2.81 (1.32-5.97)	0.007	3.10 (1.69-5.67)	0.000	3.24 (1.88-5.60)	0.000
	Non-ICUAC	1.96 (0.67-5.75)	0.22	1.51 (0.59-3.86)	0.39	1.92 (0.83-4.41)	0.13
Bacteremia	ICUAC	1.05 (0.45-2.43)	0.92	1.28 (0.67-2.46)	0.46	1.50 (0.83-2.72)	0.18
	Non-ICUAC	1.03 (0.33-3.24)	0.96	0.83 (0.31-2.26)	0.72	1.21 (0.53-2.76)	0.65
Total parenteral nutrition	ICUAC	0.99 (0.48-2.01)	0.97	0.89 (0.50-1.57)	0.68	0.93 (0.56-1.57)	0.80
	Non-ICUAC	1.40 (0.51-3.85)	0.52	1.43 (0.62-3.30)	0.41	1.54 (0.72-3.30)	0.26
Prior surgery	ICUAC	0.82 (0.34-1.99)	0.66	0.85 (0.42-1.71)	0.65	0.79 (0.42-1.49)	0.46
	Non-ICUAC	0.79 (0.18-3.49)	0.75	0.80 (0.24-2.69)	0.72	0.81 (0.28-2.35)	0.70
Neutropenia	ICUAC	1.93 (0.74-5.04)	0.18	2.11 (0.94-4.73)	0.07	2.45 (1.14-5.26)	0.02
	Non-ICUAC	0.93 (0.21-4.13)	0.93	1.88 (0.69-5.11)	0.21	1.65 (0.62-4.40)	0.32
Immunosuppressive therapy	ICUAC	0.45 (0.06-3.31)	0.43	0.89 (0.28-2.87)	0.85	0.73 (0.23-2.35)	0.60
	Non-ICUAC	0.87 (0.11-6.58)	0.89	1.22 (0.29-5.23)	0.79	0.94 (0.22-3.97)	0.93
Urine catheter placement	ICUAC	2.59 (0.99-6.74)	0.05	2.21 (1.07-4.57)	0.03	3.13 (1.54-6.39)	0.002
	Non-ICUAC	2.65 (0.94-7.46)	0.07	2.79 (1.19-6.54)	0.02	2.59 (1.21-5.57)	0.01
CVC placement	ICUAC	1.92 (0.86-4.30)	0.11	1.56 (0.85-2.88)	0.15	1.59 (0.91-2.78)	0.10
	Non-ICUAC	1.40 (0.51-3.85)	0.52	1.55 (0.67-3.58)	0.31	1.68 (0.78-3.58)	0.18
Prior fungal therapy	ICUAC	0.69 (0.09-5.03)	0.71	0.35 (0.05-2.53)	0.30	0.52 (0.13-2.13)	0.36
	Non-ICUAC	0.04 (0.00-319.64)	0.49	0.80 (0.11-5.98)	0.83	0.72 (0.10-5.32)	0.75
CVC removal	ICUAC	0.29 (0.07-1.25)	0.10	0.59 (0.24-1.45)	0.26	0.57 (0.25-1.30)	0.18
	Non-ICUAC	0.77 (0.18-3.24)	0.73	0.95 (0.30-2.99)	0.93	0.88 (0.31-2.47)	0.81
Azole monotherapy	ICUAC	0.22 (0.05-0.92)	0.04	0.32 (0.13-0.82)	0.02	0.35 (0.16-0.78)	0.01
	Non-ICUAC	0.55 (0.15-1.94)	0.35	0.99 (0.40-2.44)	0.99	0.72 (0.30-1.69)	0.45
Non-azole therapy	ICUAC	0.33 (0.15-0.75)	0.008	0.61 (0.34-1.08)	0.09	0.71 (0.42-1.20)	0.20
	Non-ICUAC	0.28 (0.08-0.99)	0.05	0.32 (0.12-0.86)	0.02	0.55 (0.25-1.22)	0.14
Lack of antifungal therapy	ICUAC	6.54 (3.05-14.04)	0.000	3.79 (2.13-6.76)	0.000	3.25 (1.92-5.51)	0.000

	Non-ICUAC	5.69 (2.00-16.20)	0.001	3.40 (1.45-7.94)	0.005	2.89 (1.33-6.28)	0.007
Fluconazole resistance	ICUAC	3.29 (0.44-24.43)	0.24	3.29 (0.44-24.43)	0.24	3.29 (0.44-24.43)	0.24
	Non-ICUAC	0.05 (0.00-1,318,766.81)	0.73	0.05 (0.00-33,105.24)	0.66	1.92 (0.26-14.23)	0.52
Micafungin resistance	ICUAC	-	-	-	-	-	-
	Non-ICUAC	0.05 (0.00-1,318,766.81)	0.73	0.05 (0.00-33,105.24)	0.66	0.05 (0.00-33,105.24)	0.66

Abbreviations: ICUAC, intensive care unit associated candidemia; OR, odds ratio; 95% CI, 95% confidence interval; ACCI, age-adjusted Charlson comorbidity index; CVC, central venous catheter.

Supplementary Table S2C. Univariate analysis of predictive factors related to 7-, 30- and 90-day mortalities of ICUAC and non-ICUAC due to *Candida glabrata*

		7-day mortality		30-day mortality		90-day mortality	
Variables	Setting	OR (95% CI)	*p*-Value	OR (95% CI)	*p*-Value	OR (95% CI)	*p*-Value
Aged 65 or older	ICUAC	5.23 (1.21-22.70)	0.03	2.76 (1.12-6.82)	0.03	2.12 (0.98-4.61)	0.06
	Non-ICUAC	0.47 (0.13-1.77)	0.27	0.42 (0.18-0.98)	0.05	0.63 (0.29-1.37)	0.25
Male	ICUAC	0.61 (0.25-1.49)	0.28	0.55 (0.26-1.14)	0.11	0.53 (0.27-1.05)	0.07
	Non-ICUAC	3.62 (0.75-17.44)	0.11	2.27 (0.93-5.57)	0.07	2.31 (1.05-5.11)	0.04
Community onset	ICUAC	-	-	-	-	-	-
	Non-ICUAC	0.03 (0.00-22.17)	0.30	0.63 (0.21-1.85)	0.40	0.76 (0.31-1.87)	0.55
Diabetes mellitus	ICUAC	1.14 (0.43-3.00)	0.79	0.86 (0.37-2.01)	0.73	0.89 (0.41-1.92)	0.77
	Non-ICUAC	2.56 (0.69-9.53)	0.16	0.92 (0.37-2.25)	0.85	1.25 (0.58-2.67)	0.57
Liver disease	ICUAC	2.83 (1.01-7.90)	0.05	2.24 (0.91-5.53)	0.08	1.91 (0.78-4.63)	0.15
	Non-ICUAC	0.88 (0.11-7.00)	0.90	1.59 (0.54-4.71)	0.40	1.56 (0.59-4.10)	0.37
Chronic kidney disease	ICUAC	2.76 (1.08-7.05)	0.03	2.04 (0.90-4.63)	0.09	1.91 (0.88-4.11)	0.10
	Non-ICUAC	2.41 (0.50-11.61)	0.27	0.77 (0.18-3.29)	0.72	1.19 (0.41-3.43)	0.75
Chronic obstructive pulmonary disease	ICUAC	1.49 (0.49-4.48)	0.48	1.57 (0.64-3.86)	0.33	1.38 (0.57-3.34)	0.48
	Non-ICUAC	1.50 (0.19-11.98)	0.70	1.13 (0.26-4.82)	0.87	1.80 (0.62-5.20)	0.28
Congestive heart failure	ICUAC	0.49 (0.07-3.68)	0.49	1.11 (0.33-3.66)	0.87	1.18 (0.42-3.36)	0.75
	Non-ICUAC	0.05 (0.00-22,269,423,208.21)	0.83	0.05 (0.00-419,209.60)	0.71	0.05 (0.00-28,844.78)	0.66
Myocardial infarction	ICUAC	1.34 (0.31-5.80)	0.70	1.40 (0.42-4.65)	0.58	1.15 (0.35-3.78)	0.82
	Non-ICUAC	6.31 (1.30-30.51)	0.02	2.14 (0.50-9.15)	0.31	1.47 (0.35-6.22)	0.60
Cerebro-vascular disease or transient ischemic attack	ICUAC	1.45 (0.55-3.81)	0.45	1.51 (0.69-3.32)	0.31	1.23 (0.57-2.64)	0.60
	Non-ICUAC	0.04 (0.00-735.11)	0.53	0.94 (0.22-4.01)	0.93	1.58 (0.55-4.55)	0.40
Dementia	ICUAC	1.45 (0.34-6.29)	0.62	1.74 (0.52-5.77)	0.37	1.17 (0.36-3.83)	0.80
	Non-ICUAC	2.35 (0.29-18.82)	0.42	2.35 (0.55-10.08)	0.25	0	0.08
Solid tumor	ICUAC	2.96 (1.19-7.40)	0.02	2.02 (0.92-4.46)	0.08	1.66 (0.77-3.58)	0.20

	Non-ICUAC	1.00 (0.27-3.72)	1.00	1.07 (0.46-2.51)	0.87	1.10 (0.52-2.36)	0.80
Hematologic malignancies	ICUAC	0.88 (0.12-6.61)	0.90	1.27 (0.30-5.37)	0.74	1.99 (0.60-6.56)	0.26
	Non-ICUAC	1.65 (0.21-13.18)	0.64	3.15 (1.06-9.34)	0.04	2.55 (0.88-7.37)	0.08
ACCI more than 5	ICUAC	2.84 (1.02-7.91)	0.05	1.83 (0.86-3.90)	0.12	1.40 (0.70-2.81)	0.34
	Non-ICUAC	1.60 (0.33-7.71)	0.56	1.09 (0.43-2.80)	0.85	0.96 (0.42-2.20)	0.93
Severe sepsis	ICUAC	1.50 (0.60-3.74)	0.38	1.33 (0.63-2.79)	0.45	1.79 (0.90-3.57)	0.10
	Non-ICUAC	1.93 (0.48-7.70)	0.35	2.25 (0.94-5.36)	0.07	2.69 (1.26-5.74)	0.01
Bacteremia	ICUAC	1.56 (0.63-3.87)	0.34	1.61 (0.77-3.37)	0.21	1.53 (0.76-3.09)	0.23
	Non-ICUAC	1.46 (0.30-7.04)	0.64	2.53 (1.03-6.21)	0.04	2.97 (1.37-6.45)	0.006
Total parenteral nutrition	ICUAC	1.08 (0.43-2.69)	0.87	0.95 (0.45-2.02)	0.90	1.04 (0.52-2.08)	0.91
	Non-ICUAC	4.19 (1.05-16.77)	0.04	2.81 (1.21-6.50)	0.02	2.45 (1.17-5.15)	0.02
Prior surgery	ICUAC	0.79 (0.26-2.38)	0.67	0.91 (0.39-2.13)	0.83	1.12 (0.52-2.41)	0.78
	Non-ICUAC	0.04 (0.00-271.15)	0.48	1.12 (0.33-3.80)	0.85	0.85 (0.26-2.83)	0.80
Neutropenia	ICUAC	0.05 (0.00-961.68)	0.55	0.61 (0.08-4.53)	0.63	0.59 (0.08-4.36)	0.61
	Non-ICUAC	1.05 (0.13-8.39)	0.96	1.53 (0.45-5.18)	0.49	1.78 (0.61-5.14)	0.29
Immunosuppressive therapy	ICUAC	-	-	-	-	-	-
	Non-ICUAC	2.17 (0.27-17.39)	0.46	1.69 (0.40-7.24)	0.48	1.24 (0.29-5.23)	0.77
Urine catheter placement	ICUAC	3.42 (0.79-14.82)	0.10	4.40 (1.32-14.65)	0.02	3.07 (1.17-8.01)	0.02
	Non-ICUAC	1.36 (0.37-5.08)	0.64	1.19 (0.51-2.78)	0.69	1.57 (0.75-3.31)	0.23
CVC placement	ICUAC	2.24 (0.65-7.68)	0.20	1.38 (0.59-3.24)	0.46	1.41 (0.64-3.13)	0.40
	Non-ICUAC	2.16 (0.54-8.62)	0.28	1.89 (0.79-4.50)	0.15	1.36 (0.64-2.88)	0.42
Prior fungal therapy	ICUAC	1.18 (0.16-8.83)	0.87	0.57 (0.08-4.21)	0.58	0.49 (0.07-3.64)	0.49
	Non-ICUAC	1.65 (0.21-13.18)	0.64	1.53 (0.36-6.54)	0.57	1.96 (0.59-6.51)	0.27
CVC removal	ICUAC	0.23 (0.03-1.78)	0.16	0.50 (0.15-1.70)	0.27	0.78 (0.29-2.09)	0.62
	Non-ICUAC	0.03 (0.00-1,101.52)	0.53	0.75 (0.16-3.42)	0.71	1.04 (0.29-3.74)	0.95
Azole monotherapy	ICUAC	0.04 (0.00-9.59)	0.25	0.62 (0.19-2.05)	0.43	0.56 (0.17-1.84)	0.34
	Non-ICUAC	0.04 (0.00-271.15)	0.48	0.79 (0.18-3.39)	0.75	0.61 (0.14-2.56)	0.50
Non-azole therapy	ICUAC	0.08 (0.01-0.62)	0.02	0.27 (0.10-0.71)	0.008	0.45 (0.21-0.97)	0.04
	Non-ICUAC	0.01 (0.00-2.47)	0.10	0.49 (0.21-1.13)	0.10	0.70 (0.33-1.48)	0.35

Lack of antifungal therapy	ICUAC	22.82 (3.03-171.75)	0.002	3.90 (1.71-8.89)	0.001	2.74 (1.33-5.62)	0.006
	Non-ICUAC	289.97 (0.27-310,796.47)	0.11	2.40 (1.04-5.57)	0.04	1.79 (0.84-3.83)	0.13
Fluconazole resistance	ICUAC	2.44 (0.70-8.43)	0.16	3.58 (1.34-9.57)	0.01	3.58 (1.34-9.57)	0.01
	Non-ICUAC	2.56 (0.32-20.50)	0.38	0.99 (0.13-7.35)	0.99	1.47 (0.35-6.21)	0.60
Micafungin resistance	ICUAC	0.05 (0.00-55,430.22)	0.67	0.05 (0.00-3,684.65)	0.60	0.05 (0.00-5,279.06)	0.61
	Non-ICUAC	0.05 (0.00-22,269,423,208.21)	0.83	3.84 (0.51-28.72)	0.19	3.84 (0.51-28.72)	0.19

Abbreviations: ICUAC, intensive care unit associated candidemia; OR, odds ratio; 95% CI, 95% confidence interval; ACCI, age-adjusted Charlson comorbidity index; CVC, central venous catheter.

Supplementary Table S2D. Univariate analysis of predictive factors related to 7-, 30- and 90-day mortalities of candidemia patients due to *Candida parapsilosis*

		7-day mortality		30-day mortality		90-day mortality	
Variables	Setting	OR (95% CI)	*p*-Value	OR (95% CI)	*p*-Value	OR (95% CI)	*p*-Value
Aged 65 or older	ICUAC	11.46 (1.45-90.62)	0.02	3.82 (1.36-10.75)	0.01	3.58 (1.37-9.37)	0.009
	Non-ICUAC	0.87 (0.12-6.19)	0.89	1.16 (0.26-5.17)	0.85	1.47 (0.35-6.16)	0.60
Male	ICUAC	0.28 (0.08-1.01)	0.05	0.51 (0.20-1.29)	0.15	0.61 (0.25-1.50)	0.28
	Non-ICUAC	1.00 (0.10-9.61)	1.00	0.82 (0.16-4.23)	0.81	0.99 (0.20-4.93)	0.99
Community onset	ICUAC	-	-	-	-	-	-
	Non-ICUAC	0.04 (0.00-5,311.54)	0.59	0.04 (0.00-233.28)	0.46	0.04 (0.00-115.29)	0.42
Diabetes mellitus	ICUAC	0.35 (0.04-2.74)	0.32	0.59 (0.17-2.02)	0.40	0.73 (0.24-2.17)	0.57
	Non-ICUAC	2.58 (0.36-18.31)	0.34	3.52 (0.79-15.75)	0.10	2.71 (0.68-10.84)	0.16
Liver disease	ICUAC	6.41 (0.79-52.13)	0.08	6.41 (0.79-52.13)	0.08	6.41 (0.79-52.13)	0.08
	Non-ICUAC	0.04 (0.00-165,482.07)	0.69	0.04 (0.00-3,361.80)	0.58	0.04 (0.00-2,165.81)	0.57
Chronic kidney disease	ICUAC	0.74 (0.09-5.87)	0.78	0.37 (0.05-2.75)	0.33	0.68 (0.16-2.92)	0.60
	Non-ICUAC	0.04 (0.00-23,302.06)	0.64	1.19 (0.14-9.89)	0.87	1.04 (0.13-8.47)	0.97
Chronic obstructive pulmonary disease	ICUAC	0.62 (0.08-4.88)	0.65	1.12 (0.32-3.86)	0.86	1.42 (0.48-4.26)	0.53
	Non-ICUAC	0.05 (0.00-1,370,187,406.67)	0.80	4.91 (0.59-40.90)	0.14	4.91 (0.59-40.90)	0.14
Congestive heart failure	ICUAC	0.04 (0.00-593.85)	0.52	0.53 (0.07-4.02)	0.54	1.02 (0.24-4.40)	0.98
	Non-ICUAC	0.05 (0.00-1,370,187,406.67)	0.80	4.91 (0.59-40.90)	0.14	4.32 (0.53-35.20)	0.17
Myocardial infarction	ICUAC	3.19 (0.67-15.11)	0.14	1.74 (0.40-7.61)	0.46	1.54 (0.36-6.63)	0.57
	Non-ICUAC	5.75 (0.60-55.37)	0.13	2.80 (0.34-23.29)	0.34	2.28 (0.28-18.59)	0.44
Cerebro-vascular disease or transient ischemic attack	ICUAC	0.39 (0.05-3.11)	0.38	0.39 (0.09-1.71)	0.21	0.77 (0.26-2.31)	0.64
	Non-ICUAC	0.04 (0.00-18,935.46)	0.63	1.20 (0.14-9.96)	0.87	1.08 (0.13-8.81)	0.94
Dementia	ICUAC	0.05 (0.00-6,017,280.36)	0.75	3.14 (0.41-24.04)	0.27	3.14 (0.41-24.04)	0.27

	Non-ICUAC	0.05 (0.00-25,331,786,021,976.70)	0.86	0.05 (0.00-3,174,566,202.89)	0.81	0.05 (0.00-334,821,302.42)	0.79
Solid tumor	ICUAC	2.20 (0.57-8.52)	0.25	1.70 (0.56-5.19)	0.35	1.53 (0.51-4.60)	0.45
	Non-ICUAC	1.13 (0.16-8.02)	0.90	0.85 (0.19-3.79)	0.83	1.13 (0.28-4.51)	0.86
Hematologic malignancies	ICUAC	-	-	-	-	-	-
	Non-ICUAC	0.05 (0.00-25,331,786,021,977.00)	0.86	11.24 (1.31-96.25)	0.03	11.24 (1.31-96.25)	0.03
ACCI more than 5	ICUAC	1.45 (0.41-5.13)	0.57	1.12 (0.42-3.00)	0.82	1.23 (0.49-3.09)	0.66
	Non-ICUAC	4.53 (0.47-43.58)	0.19	3.87 (0.75-19.98)	0.11	4.95 (1.00-24.64)	0.05
Severe sepsis	ICUAC	3.18 (0.92-11.01)	0.07	2.07 (0.80-5.35)	0.13	2.21 (0.90-5.43)	0.08
	Non-ICUAC	2.82 (0.29-27.09)	0.37	6.67 (1.49-29.84)	0.01	6.13 (1.43-26.33)	0.02
Bacteremia	ICUAC	0.04 (0.00-38.86)	0.35	0.52 (0.12-2.27)	0.39	0.75 (0.22-2.57)	0.65
	Non-ICUAC	0.04 (0.00-1,350,428.16)	0.72	0.04 (0.00-14,151.00)	0.63	0.04 (0.00-4,543.68)	0.60
Total parenteral nutrition	ICUAC	1.21 (0.35-4.18)	0.77	1.20 (0.48-3.02)	0.70	1.22 (0.51-2.92)	0.66
	Non-ICUAC	1.46 (0.21-10.36)	0.71	3.66 (0.71-18.89)	0.12	2.46 (0.59-10.28)	0.22
Prior surgery	ICUAC	0.74 (0.16-3.48)	0.70	0.84 (0.28-2.55)	0.75	1.01 (0.36-2.77)	0.99
	Non-ICUAC	0.04 (0.00-63,689.70)	0.66	0.04 (0.00-1,480.28)	0.55	0.04 (0.00-662.96)	0.52
Neutropenia	ICUAC	1.67 (0.21-13.19)	0.63	2.42 (0.55-10.64)	0.24	2.21 (0.51-9.62)	0.29
	Non-ICUAC	0.04 (0.00-63,689.70)	0.66	0.04 (0.00-1,480.28)	0.55	0.04 (0.00-662.96)	0.52
Immunosuppressive therapy	ICUAC	-	-	-	-	-	-
	Non-ICUAC	7.63 (0.79-73.79)	0.08	3.95 (0.47-32.87)	0.20	3.26 (0.40-26.55)	0.27
Urine catheter placement	ICUAC	2.24 (0.48-10.57)	0.31	3.20 (0.92-11.08)	0.07	3.77 (1.10-12.89)	0.04
	Non-ICUAC	0.46 (0.05-4.43)	0.50	1.77 (0.40-7.92)	0.45	2.22 (0.53-9.31)	0.27
CVC placement	ICUAC	3.23 (0.69-15.21)	0.14	2.35 (0.84-6.60)	0.11	2.80 (1.01-7.71)	0.05
	Non-ICUAC	0.26 (0.03-2.52)	0.25	0.59 (0.13-2.61)	0.48	0.76 (0.19-3.05)	0.70
Prior fungal therapy	ICUAC	1.31 (0.17-10.38)	0.80	0.65 (0.09-4.88)	0.68	0.56 (0.08-4.20)	0.57
	Non-ICUAC	29.50 (1.84-471.61)	0.02	29.50 (1.84-471.61)	0.02	29.50 (1.84-471.61)	0.02
CVC removal	ICUAC	0.18 (0.02-1.43)	0.10	0.31 (0.08-1.13)	0.08	0.23 (0.07-0.84)	0.03
	Non-ICUAC	0.02 (0.00-232,409.72)	0.64	0.58 (0.05-6.47)	0.66	0.36 (0.04-3.52)	0.38
Azole monotherapy	ICUAC	0.02 (0.00-2.77)	0.12	0.26 (0.09-0.80)	0.02	0.38 (0.15-0.99)	0.05

	Non-ICUAC	0.27 (0.03-2.61)	0.26	0.60 (0.13-2.67)	0.50	0.78 (0.19-3.11)	0.72
Non-azole therapy	ICUAC	0.03 (0.00-9.02)	0.23	0.44 (0.13-1.52)	0.20	0.39 (0.11-1.32)	0.13
	Non-ICUAC	0.04 (0.00-4,604.24)	0.59	0.04 (0.00-220.03)	0.46	0.04 (0.00-118.54)	0.43
Lack of antifungal therapy	ICUAC	612.80 (0.18-2,062,854.44)	0.12	9.23 (3.42-24.88)	<0.001	7.64 (3.03-19.28)	<0.001
	Non-ICUAC	7.29 (0.76-70.09)	0.09	3.46 (0.77-15.50)	0.11	2.71 (0.68-10.90)	0.16
Fluconazole resistance	ICUAC	0.04 (0.00-593.85)	0.52	0.62 (0.08-4.64)	0.64	0.56 (0.08-4.20)	0.57
	Non-ICUAC	-	-	-	-	-	-
Micafungin resistance	ICUAC	-	-	-	-	-	-
	Non-ICUAC	-	-	-	-	-	-

Abbreviations: ICUAC, intensive care unit associated candidemia; OR, odds ratio; 95% CI, 95% confidence interval; ACCI, age-adjusted Charlson comorbidity index; CVC, central venous catheter.
