# Supplementary material for: Dynamics and Predictors of Mortality Due to Candidemia Caused by Different Candida Species: Comparison of Intensive Care Unit-Associated Candidemia (ICUAC) and Non-ICUAC
Source: J Fungi (Basel). 2021 Jul 24;7(8):597. doi: 10.3390/jof7080597 (PMC8397010; doi:10.3390/jof7080597)
Supplement: Supplementary file 1 [file jof-07-00597-s001.zip › Supplementary Table S3.pdf]

Supplementary Table S3. Multivariate analysis of predictive factors related to 7-, 30- and 90-day mortalities of non-ICUAC by the four common *Candida* species

	7-day		30-day		90-day	
Species and variables[a]	OR (95% CI)	*p*-Value	OR (95% CI)	*p*-Value	OR (95% CI)	*p*-Value
C. albicans						
Lack of antifungal therapy	34.64 (7.86-152.68)	0.006	10.49 (4.68-23.51)	<0.001	3.97 (2.32-6.79)	<0.001
CVC placement	-	-	-	-	2.05 (1.20-3.51)	0.009
Urinary catheter placement	-	-	2.70 (1.25-5.84)	0.01	1.79 (1.07-2.99)	0.03
Severe sepsis	-	-	-	-	2.57 (1.49-4.43)	0.001
Azole monotherapy	0.06 (0.01-0.44)	0.01	0.26 (0.11-0.62)	0.002	0.33 (0.19-0.59)	<0.001
CVC removal	-	-	-	-	0.34 (0.15-0.80)	0.01
C. tropicalis						
Lack of antifungal therapy	7.74 (2.59-23.17)	0.04	4.54 (1.87-11.00)	0.001	6.02 (2.45-14.81)	<0.001
Urinary catheter placement	3.91 (1.33-11.52)	0.01	3.68 (1.52-8.91)	0.004	3.69 (1.62-8.43)	0.002
Diabetes mellitus	-	-	-	-	0.21 (0.07-0.65)	0.007
C. glabrata						
Lack of antifungal therapy	-	-	5.02 (1.82-13.88)	0.002	-	-
Myocardial infarction	5.71 (1.17-27.80)	0.03	-	-	-	-
Total parental nutrition	-	-	2.74 (1.17-6.44)	0.02	2.35 (1.09-5.03)	0.03
Bacteremia	-	-	4.22 (1.44-12.34)	0.009	3.12 (1.40-6.98)	0.005
Male	-	-	-	-	2.48 (1.12-5.51)	0.03
Aged 65 or older	-	-	0.42 (0.18-0.98)	0.05	-	-
C. parapsilosis						

Prior fungal therapy	20.83 (1.28-339.02)	0.03	114.11 (5.07-2,566.86)	0.03	221.43 (7.17-6,835.92)	0.002
Severe sepsis	-	-	7.92 (1.58-39.62)	0.01	6.73 (1.39-32.66)-	0.02

Abbreviations: ICUAC, intensive care unit-associated candidemia; OR, odds ratio; 95% CI, 95% confidence interval; CVC, central venous catheter.

[a]Only the variables that were statistically significant (P <0.05) are listed.
